
JOURNAL INFORMATION
==============================
NLM Title Abbreviation: ScientificWorldJournal
Journal ID: ScientificWorldJournal
NLM Journal ID: 101131163
Journal ID: TSWJ

The Scientific World Journal

ISSN: 2356-6140
EISSN: 1537-744X
Publisher: Wiley

ARTICLE INFORMATION
==============================
PMCID: PMC5763940
PMID: 20062952
DOI: 10.1100/tsw.2010.11
Article ID: 814930
Article version: 1
Subjects: Clinical Images

Rubeosis Faciei Diabeticorum: a Common, but Often Unnoticed, Clinical Manifestation of Diabetes Mellitus

Namazi M. R. 1
Jorizzo J. L. 2
Fallahzadeh M. K. 1 *
1 Dermatology Department, Shiraz University of Medical Sciences, Shiraz, Iran
2 Dermatology Department, Wake Forest University Health Sciences, Winston-Salem, North Carolina, USA
*M. K. Fallahzadeh: kazem.fa@gmail.comAcademic Editor: Daniel Batlle

Publication date: 2010
Electronic publication date: 2010 Jan 8
Volume: 10
First page: 70
Last page: 71
Received 2009 Nov 3; Revised 2009 Nov 15; Accepted 2009 Dec 16
Copyright: Copyright © 2010 M. R. Namazi et al.
Copyright year: 2010
License: This is an open access article distributed under the Creative Commons Attribution License, which permits unrestricted use, distribution, and reproduction in any medium, provided the original work is properly cited.
License URL: https://creativecommons.org/licenses/by/3.0/

Keywords: rubeosis faciei diabeticorum, diabetes mellitus, microangiopathy, hyperglycemia

==============================
Clinical Image
*TheScientificWorldJOURNAL* (2010) 10, 70–71
TSW Clinical Images
ISSN 1537-744X; DOI 10.1100/tsw.2010.11

TheScientificWorldJOURNAL
www.thescientificworld.com

### Rubeosis Faciei Diabeticorum: a Common, but Often Unnoticed, Clinical Manifestation of Diabetes Mellitus

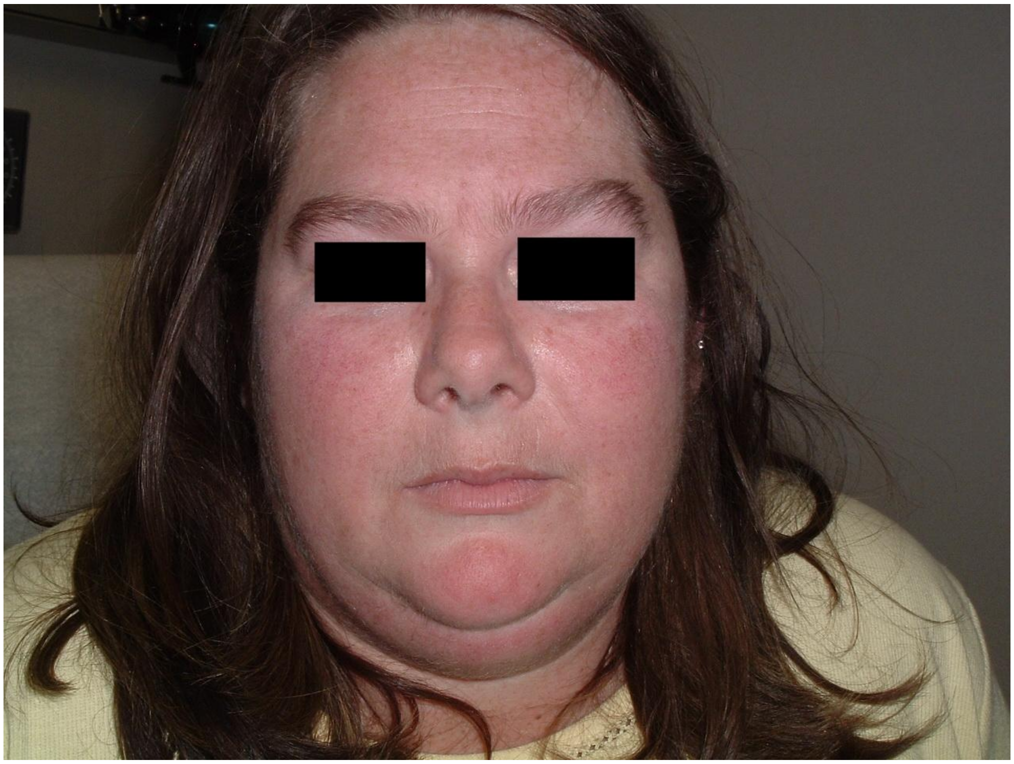

**FIGURE.** Rubeosis faciei diabeticorum in a patient with diabetes mellitus.

M.R. Namazi[1], J.L. Jorizzo[2], and M.K. Fallahzadeh[1,]*

*[1]Dermatology Department, Shiraz University of Medical Sciences, Shiraz, Iran;*
*[2]Dermatology Department, Wake Forest University Health Sciences, Winston-Salem, North Carolina*

**E-mail:** kazem.fa@gmail.com

*Received November 3, 2009; Revised November 15, 2009; Accepted December 16, 2009; Published January 8, 2010*

**KEYWORDS**: rubeosis faciei diabeticorum, diabetes mellitus, microangiopathy, hyperglycemia

*Corresponding author.
©2010 with author.
Published by TheScientificWorld; www.thescientificworld.com

Rubeosis, also named rubeosis facei and rubeosis faciei diabeticorum, occurs in up to 59% of hospitalized patients with diabetes mellitus[1] and is often a sign of suboptimal glycemic control[2]. In diabetics, hyperglycemia could lead to sluggish microcirculation, which becomes clinically evident by facial venous dilatation[3]. Rubeosis signifies microangiopathy, necessitating the evaluation of the patients for other more important microangiopathies, such as retinopathy[2].

Strict glycemic control is the mainstay of treatment for this reddish complexion[2].

### REFERENCES

1. Gitelson, S. and Wertheimer-Kaplinski, N. (1965) Color of the face in diabetes mellitus; observations on a group of patients in Jerusalem. *Diabetes* **14,** 201–208.
2. Paron, N.G. and Lambert, P.W. (2000) Cutaneous manifestations of diabetes mellitus. *Prim. Care* **27**, 71–83.
3. Huntley, A. (1995) Diabetes mellitus: review. *Dermatol. Online J.* **1(2).** Address: http://dermatology.cdlib.org/DOJvol1num2/diabetes/dmreview.html [accessed: November 12, 2009].

**This article should be cited as follows:**

Namazi, M.R., Jorizzo, J.L., and Fallahzadeh, M.K. (2010) Rubeosis faciei diabeticorum: a common, but often unnoticed, clinical manifestation of diabetes mellitus. *TheScientificWorldJOURNAL:* TSW Clinical Images **10**, 70–71. DOI 10.1100/tsw.2010.11.
